# The effects of poaching and habitat structure on anti-predator behavioral strategies: A guanaco population in a high cold desert as case study

**DOI:** 10.1371/journal.pone.0184018

**Published:** 2017-08-31

**Authors:** Flavio Cappa, Valeria Campos, Stella Giannoni, Natalia Andino

**Affiliations:** 1 INTERBIODES (Interacciones Biológicas del Desierto), Facultad Ciencias Exactas, Físicas y Naturales (Universidad Nacional de San Juan), San Juan, Argentina; 2 CIGEOBIO-CONICET (Centro de Investigaciones de la Geósfera y Biósfera – Consejo Nacional de Investigaciones Científicas y Técnicas), San Juan, Argentina; 3 Instituto y Museo de Ciencias Naturales, Facultad Ciencias Exactas, Físicas y Naturales, Universidad Nacional de San Juan, San Juan, Argentina; Sichuan University, CHINA

## Abstract

The effects of poaching on wildlife have been widely studied in conservation biology and can be heterogeneous, particularly on ungulates. These effects can be estimated through different methodologies whose use depends on several conditions such as Flight-initiation distance (FID). Our objectives were: 1- to evaluate whether poaching affects the FID and group structure of a guanaco (*Lama guanicoe*) population in a high cold desert in San Juan (Argentina); 2- to assess whether habitat structure (slope and vegetation cover) influences FID and group structure in this population. The study area included a site with poaching (unprotected area), and a site without poaching (protected area).

We recorded 100 groups of guanacos: 70 in the protected and 30 in the unprotected area. FID and group size were greater in the unprotected than in the protected area, whereas proportions of group categories (with offspring, without offspring and solitary) were similar between areas. Besides, in relation to habitat structure, FID increased when vegetation cover decreased. On the other hand, FID and group size were not affected by slope. Our study shows that guanacos respond to poaching pressure as do other ungulate species, and that other factors such as vegetation cover also affect this behavior. Managers should be aware when interpreting FID due to its relation to habitat structure; the guanaco appears to assume greater risk (lower FID) in areas with high vegetation cover.

## Introduction

In an increasingly human-dominated world, it is necessary to understand the effects of human disturbances in order to achieve effective management and conservation of wildlife. Human activities such as motorized vehicle use [1], tourism [2], and legal and illegal hunting [3, 4] have a variety of direct and indirect effects on animals. Wildlife managers need to be able to identify and quantify these effects for a successful management of populations. Anthropogenic effects can be estimated through different methodologies whose use depends on several conditions, for example animal species, ease of use, and economic costs [5], among others.

Behavioral ecological methods are frequently used to assess anthropogenic stressors because ultimately a species’ behavioral response to humans will influence its ability to coexist with them. Flight-initiation distance (FID) is among behavioral responses, and is the distance between an animal and an observer at the moment the animal begins to flee [6]. FID is frequently used by managers because it is relatively simple to evaluate, non-invasive, and inexpensive. This measure is used to quantify shyness or sensitivity to human disturbances because flight is a common animal behavioral response to human presence [7, 6]. While managers acknowledge the variability in FID, they nevertheless use estimates of a species’ FID to attempt to minimize human impact. This measure is best used by comparing disturbed and undisturbed populations, where differences should be attributable to differential habituation [8, 9].

FID has been used in several ungulate species: guanacos (*Lama guanicoe*) [3, 2], impala (*Aepyceros melampus*) and greater kudu (*Tragelaphus strepsiceros*) [10], mule deer (*Odocoileus hemionus*) [11], Thomson’s gazelle (*Eudorcas thomsonii*) [12], among others. The general pattern is that ungulates exhibit longer FID when they are under hunting pressure [3], however this is not clear in guanacos, the largest ungulate inhabiting arid and semiarid ecosystems in South America. Donadio and Buskirk [3] in San Guillermo National Park, San Juan Province, found no statistical differences in FID between areas with and without poaching. Nor was poaching found to affect FID in La Payunia Provincial Reserve in Mendoza Province either [13]. Nevertheless, Malo and collaborators [2] found that the probability of neutral human encounters generated a decrease in FID in this species.

The effect of legal and illegal hunting pressure can be highly heterogeneous, which makes it important to analyze complementary biological and behavioral aspects, including FID, if we want to know the effect of a certain disturbance upon any animal population [6]. Group living is another behavioral strategy exhibited by different ungulate species in areas with high predation risk [14, 15, 16]. Increasing group size improves the likelihood of detecting predators due to the collective detection effect [17]. In a variety of species, a positive relation has been found between group size and FID, which indicates that animals in larger groups initiate flight at greater distances [18]. For this reason, group formation is important to ungulate females with offspring that are vulnerable to predation [19], and that must choose areas with low predation risk, such as inside a protected area.

The effect of human activities, like poaching, on animal behavior could be influenced by habitat structure, which can be characterized through biophysical predictors. Measures of these predictors are difficult to identify on the basis of field studies across large areas [20; 21]. Topography and vegetation structure [22, 13] can be assessed with remotely sensed data [23, 24] through a multi-scale analysis in broad areas [25, 26]. However, this information has been scarcely used for explaining ungulate behavior. Taraborelli and collaborators [13] found shorter alert distances of guanacos on steeper slopes. Estes and collaborators [27] used texture analysis of SPOT, ASTER and MODIS remote sensing images, and found them to be a good tool for understanding habitat selection by different ungulates.

The guanaco is a resource-defense polygynous species, with family groups comprising a territorial male with multiple females and their offspring [28, 29]. Towards the end of the 19th century, guanaco populations were present in nearly all Argentine biomes, occupying open woodlands and scrub-dominated areas. Currently, guanacos are abundant only in Patagonian steppes and Andean foothills [2]. There are several causes for this decline: legal and illegal hunting, competition with domestic livestock and/or exotic herbivores, and habitat loss or fragmentation by agricultural development [30, 31]. As a consequence of this, the guanaco was listed in Appendix II of the CITES (Convention on International Trade in Endangered Species) in 1978, which only permits the use of fiber from live animals [32].

Hunting of guanacos is controlled in Argentina by Law No. 20961/75, and is particularly prohibited in the province of San Juan (Law No. 6911), notwithstanding, they are poached in almost the entire Province. Based on poaching pressure and habitat destruction, in San Juan province the guanaco has been deemed a “vulnerable” species by the Secretary of State for the Environment and Sustainable Development (Resolution No. 0656-SEAyDS-11).

At a time when biodiversity loss is a major conservation concern, protected areas are the only sites where native species population conservation is expected to occur. In these areas it becomes necessary to find assessment tools and assess the factors affecting these tools. FID is a commonly used method to evaluate the poaching pressure on ungulates and other animals. In guanacos, no strong evidence has been found that FID is affected by poaching in a similar way. Due to this, our objectives were: 1- to evaluate whether poaching affects the FID and group structure (group size and kind of group) of a guanaco population in a high cold desert in San Juan province; 2- to assess whether habitat structure influences FID and group structure in this population.

## Materials and methods

### Ethics statement

This study was conducted with wild free-ranging animals and was completely observational. Research was conducted in accordance with guidelines of the Bioethics Commission, Departamento de Biología, Facultad de Ciencias Exactas, Físicas y Naturales, Universidad Nacional de San Juan, Argentina and the provincial authorities where the work occurred. Approval and permission to conduct research was granted by the Mr. Arturo Curatola owner of Don Carmelo Reserve and the Dirección de Conservación y Áreas Protegidas dependent of Secretaría de Estado, Ambiente y Desarrollo Sustentable of San Juan Province.

### Study area

**Fig 1:**
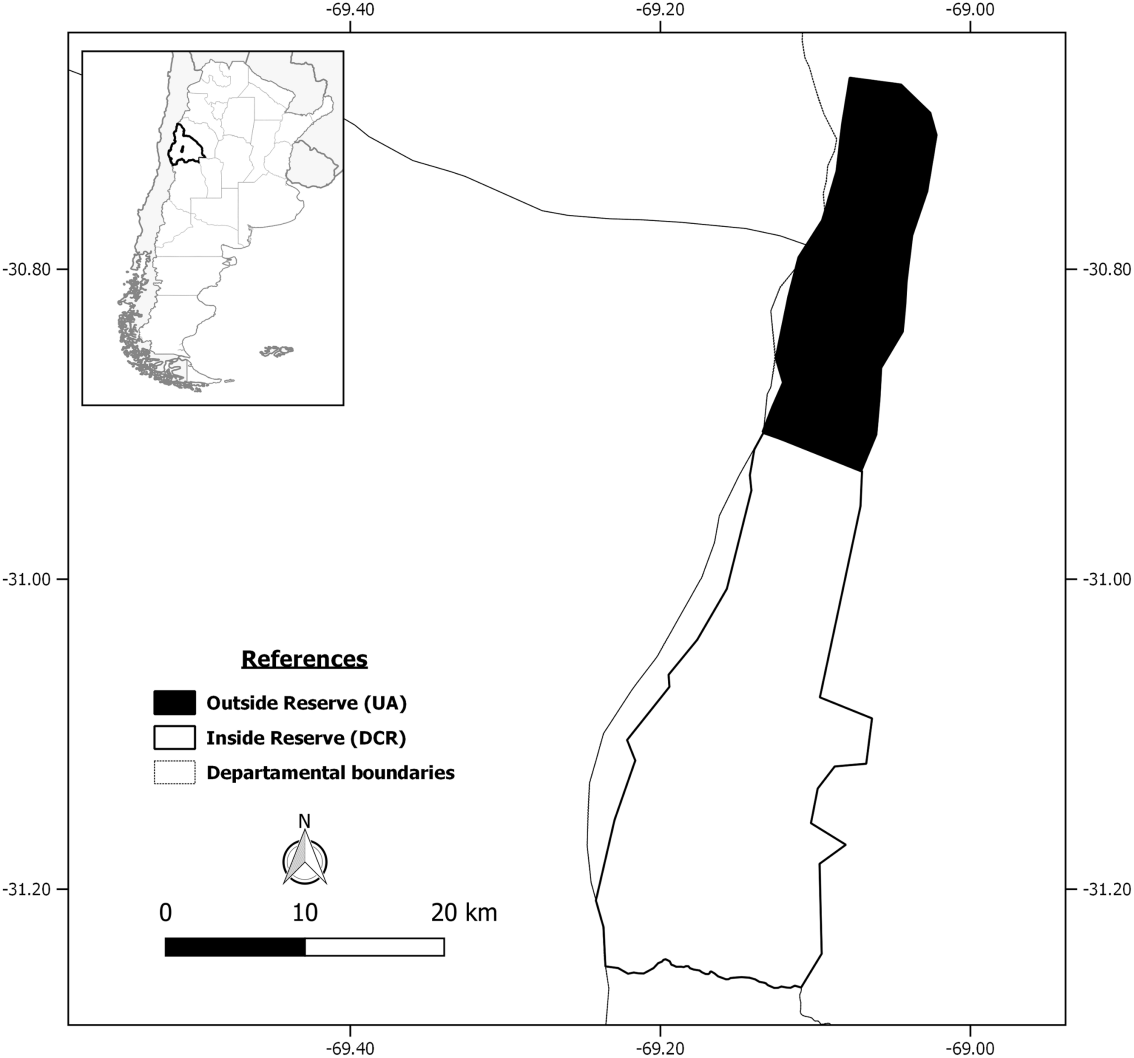
Geographic location of La Invernada Valley, San Juan, Argentina.

The study was conducted in La Invernada Valley, Ullúm department (31° 10' S, 69° 46' W), San Juan province, Argentina [33] (Fig 1). The study area lies at between 2000 and 3100 m a.s.l., and comprises sectors of the Puna Desert and Monte Desert which correspond to the Puna and Monte of hills and closed basins (Monte de Sierras y Bolsones) ecoregions respectively. The vegetation in the Puna is dominated by *Lycium chanar*, *Artemisia mendozana*, *Maihueniopsis glomerata*, *Adesmia* aff. *horrida* and *Stipa* spp., with *Lilaeopsis macloviana*, *Taraxacum officinale*, *Nastanthus agglomeratus*, *Azorella* spp. and *Adesmia pinnifolia* being less abundant [33]. The Monte is characterized by *Larrea divaricata*, *Monttea aphylla*, *Gochnatia glutinosa* and *Trycicla spinosa* [33]. In general, the area is a shrub steppe dominated by a gently undulating relief and flatlands with short xerophytic vegetation.

La Invernada Valley covers about 58000 ha and includes a protected area, Don Carmelo Reserve (Provincial decree 1220/1993), of about 40000 ha (hereafter called DCR). The remaining unprotected area (hereafter called UA, 18000 ha) is vulnerable to poaching events because there is no law enforcement. This UA stretches between the Reserve and Provincial Route No. 436. The animals can move freely into and out of DCR because this protected area is delimited by a single wire that is 15 cm above the ground. Density of guanacos in La Invernada Valley is one of the highest in the Province (4–6 ind/km^2^), and livestock are not present [34].

### Field sampling

The study was conducted in May 2010, March 2011 and February 2012. The study area was divided into the area where poaching occurs, Unprotected area (UA), and does not occur, Don Carmelo Reserve (DCR, Fig 1). We monitored the FID of guanaco groups in both areas. We used different dirt roads that run across the study area; these were travelled by four observers in a pickup truck (20–35 km/h). We used this methodology because poachers travel in pickup trucks along dirt roads seeking to shoot the animals (Secretary of State for the Environment and Sustainable Development, pers. comm.). The low vegetation and gentle slope in the study area allow a suitable visibility for monitoring the behavior of guanacos.

We identified individuals or groups based on the cohesive behavior among members [3]; thus, individuals were considered in the same group when they were at a maximum distance of 50 m from each other. We measured FID with a laser rangefinder (7x26 Bushnell ELITE 1500) from the center of the group when the first individual fled. To analyze group structure, we recorded size and age composition for each group. Categorizing family and bachelor groups at long distances is difficult without marked animals [30]; due to this, we considered three group composition categories: 1-groups with offspring; 2- groups without offspring, and 3- solitary adult. If we saw a group with the same structure (size and composition) and position as a group recorded on the previous day, we did not record this data. We treated each group as an independent sample because we travelled the dirt roads only once a day.

### Habitat structure

For habitat structure, we considered slope and heterogeneity of vegetation from remote sensing data. References used for images were: WGS 84 datum, UTM (Universal Transverse Mercator) projection; zone: Argentina 19 South. Slope angle (in degrees) was modelled on the basis of a MDE-Ar (Modelo Digital de Elevaciones de la República Argentina 30 m-resolution). This model is based on SRTM (Shuttle Radar Topography Mission, see http://www.ign.gob.ar/NuestrasActividades/Geodesia/ModeloDigitalElevaciones/Introduccion). For the analysis of vegetation, Landsat 8 OLI scenes (30-m resolution) of the study area (path 232 and row 81–82 for La Invernada Valley) acquired on 27 February 2011 (http://earthexplorer.usgs.gov/) were used. This date was selected because the images had no cloud cover. Images were rescaled to the Top Of Atmosphere (TOA) reflectance with a correction for the sun angle using coefficients provided in the product metadata file (MTL file). The SATVI green index (Soil Adjusted Total Vegetation Index) [35] was the basis for the image texture analyses to assess the heterogeneity of vegetation [26, 36]. Several window sizes were evaluated, so the different scales were represented by the extent of the moving window of an image texture measure, i.e. with 3 x 3 (0.81 ha), 5 x 5 (2.25 ha), 7 x 7 (4.41 ha) and 9 x 9 (7.29 ha) 30-m pixel moving windows.

All remote sensing variables were finally stored as separate layers in the GIS and were extracted for each group recorded. For image analysis we used Quantum GIS (Version 2.14 Essen, http://qgis.osgeo.org/) and ENVI GIS (ENVI 2004, Research Systems, Boulder, Colorado, USA).

### Statistical analyses

All analyses were performed using Generalized Linear Mixed Models (GLMMs). To assess the effect of poaching on FID, we fitted a model with FID as response variable, poaching situation as explanatory variable with two levels: with (UA) and without poaching (DCR), and with group composition categories (group with and without offspring, and solitary adult) and year (2010, 2011 and 2012) as random variables. Because the error structure was not Gaussian, in spite of the log transformation, FID was modelled as a Gamma distribution.

To assess the poaching effect on group size (response variable) we fitted a Negative Binomial distribution because the model showed overdispersion [37] (*ĉ*>1). We did not use solitary individuals for this analysis. To assess whether poaching affected the proportion of group composition categories, we fitted models for each group category with a Binomial distribution (used for proportion data). Thus, for each fitted model we included poaching situation as explanatory variable and year as random variable.

To evaluate how FID (log transformed) and group size respond to habitat structure at different spatial scales, GLMMs with a Gaussian and Negative Binomial error structure respectively [38] were fitted slope (MDE-Ar) plus each scale considered (i.e. 3 x 3, 5 x 5, 7 x 7 and 9 x 9 30-m) for SATVI mean texture measure. We fitted four models, one for each scale considered. We used poaching situation (UA and DCR) as random factor. The habitat structure measures (slope and texture measure) that were uncorrelated were included as explanatory variables (Spearman rank correlation: r < |0.7|) [39]. A backward elimination procedure was performed to remove insignificant terms without losing important information. Backward elimination started with all of the predictors in a full model. The least significant variable, i.e. the one with the largest *P* value, was removed and the model was refitted. Each subsequent step removed the least significant variable in the model until all remaining variables had individual *P* values less than 0.05. The sign of parameters having significant effects was used to interpret the results [40, 41].

All statistical analyses were carried out using R version 3.2.2 [42]. We used nlme package [43] for Gamma and Binomial distribution and glmmADMB package [44] for Negative Binomial distribution.

## Results

**Fig 2:**
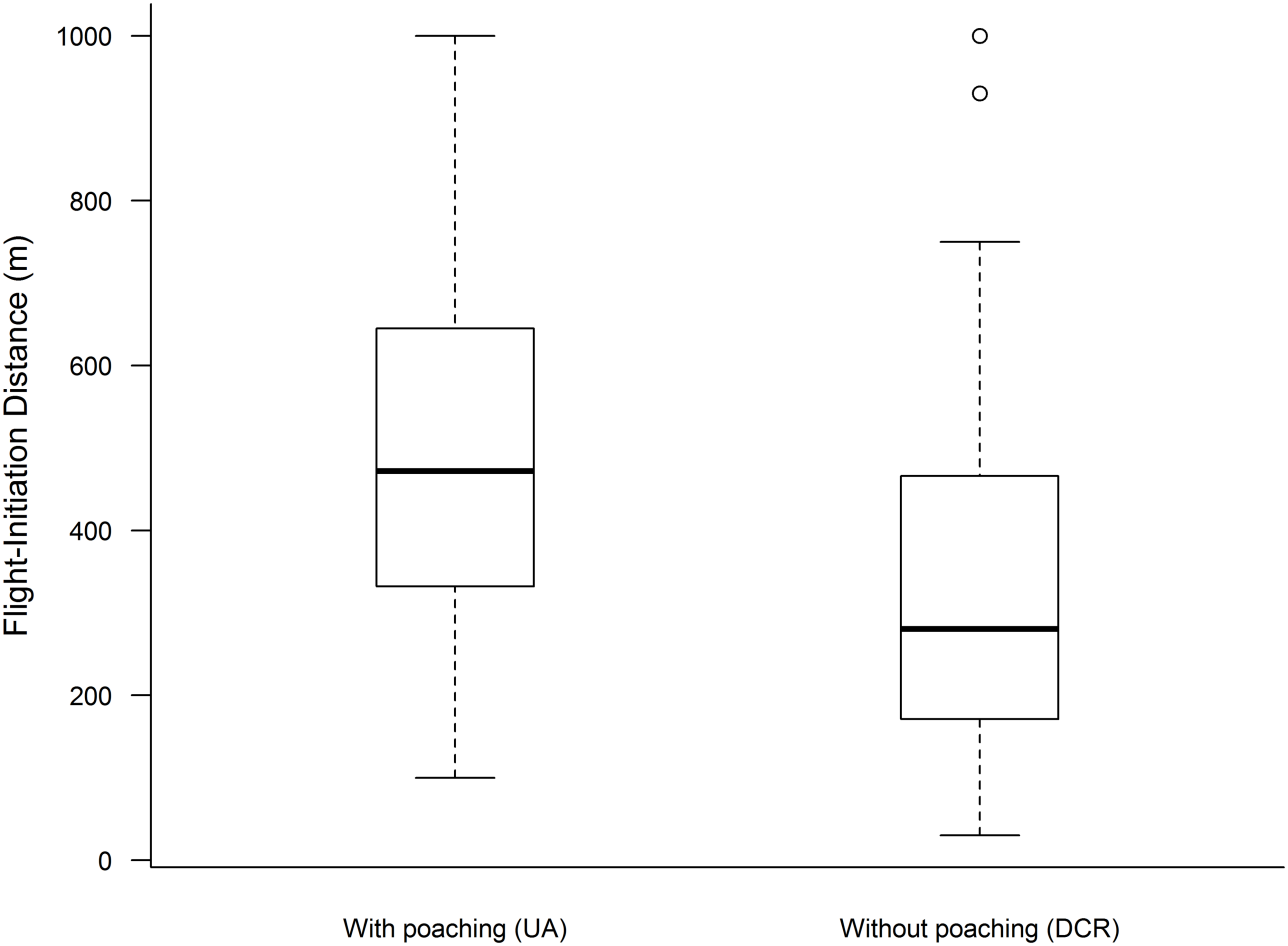
Boxplot of Flight-initiation distance (FID, in meters) in relation to the poaching situation. The horizontal bold line in the box indicates the median value of the data. The upper and lower 1 hinges of the box indicate the 75th and 25th percentiles of the data set, respectively. The ends of the vertical lines indicate the minimum and maximum data values, the points outside the ends of the whiskers are outliers.

We recorded a total of 862 guanaco observations. In the UA, we observed 30 groups (with offspring = 12, without offspring = 15, solitary = 3) and 70 groups in DCR (with offspring = 27, without offspring = 30, solitary = 13). FID was significantly higher in the UA ($\bar{x}=515.93\text{ m}$, SD = ±257.72 m) than in DCR ($\bar{x}=354.18\text{ m}$, SD = ±260.38 m; GLMM_Gamma_, t = 2.71, P = 0.007, Fig 2). Poaching also affected group size, all groups being larger (without solitary individuals) in the UA than in DCR (GLMM_Neg,Binom_, Z = -3.52, P<0.001). Notwithstanding, the number of groups for each category in the UA was less than half that in DCR, the proportion of group categories was similar in both areas, and we found no significant differences in either case (P>0.3 in all cases, Table 1).

**Table 1:** Mean group size ($\bar{x}$) with Standard Error (±SE), number of groups recorded (n) and proportion of each group category (Prop) according to the poaching situation. Significance factor for proportion of group category was <0.05.

	With poaching (UA)			Without poaching (DCR)			*P*
	$\bar{x}\pm \text{SD}$	n	Prop	$\bar{x}\pm \text{SD}$	n	Prop	
With offspring	13.16 ±10.49	12	0.4	9.46 ±5.36	27	0.38	0.85
Without offspring	15.4 ±17.83	15	0.5	6.62 ±4.19	30	0.43	0.52
Solitary	1	3	0.1	1	13	0.18	0.31

FID and group size were not affected by slope ($\bar{x}=6.90$, SD±6.05) but SATVI mean texture measure affected FID at all scales (P = 0.03, Table 2). FID decreased when SATVI mean texture measure increased, i.e. greater vegetation cover. SATVI mean texture measure did not affect group size (P>0.1) on any of the scales examined.

**Table 2:** Generalized linear mixed model for Flight-initiation distance related to image texture values from SATVI at different scales (number of pixels). Poaching situation was considered a random effect.

Fixed effect	Estimate	SE	t	P
3 x 3 moving windows (0.81 ha)				
Intercept	2531.50	919.15	2.75	0.00
Mean of SATVI	-58.45	25.46	-2.30	0.02
5 x 5 moving windows (2.25 ha)				
Intercept	2838.61	983.00	2.89	0.00
Mean of SATVI	-66.97	27.23	-2.46	0.01
7 x 7 moving windows (4.41 ha)				
Intercept	2968.36	1033.10	2.87	0.00
Mean of SATVI	-70.57	28.61	-2.46	0.01
9 x 9 moving windows (7.29 ha)				
Intercept	3094	1084.13	2.85	0.00
Mean of SATVI	-74.10	30.05	-2.46	0.01

## Discussion

We found that guanacos exhibited longer flight distances and larger group size in the UA, where they were subjected to poaching, than in DCR. Regarding the proportion of group composition categories, we found that each category was equally represented in both areas. However, we found less than half the number of groups in the UA than in DCR.

Many studies use behavioral changes as a tool for measuring disturbance; FID is frequently used to assess the effect of different types of disturbances on wildlife [7, 2]. Unlike Donadio and Buskirk [3] and Taraborelli and collaborators [13], who did not find any poaching effect on FID, we found that guanacos in DCR showed shorter FID than in the UA. This is consistent with findings for other ungulates [7, 10] which show greater FID when hunting pressure increases. Poachers used the full extent of La Invernada Valley until the Reserve was created (1993), thereafter they habitually poach only in the outside area, which probably explains our results on FID. Marino and Johnson [1] suggest that guanacos can change their tolerance to humans if stimuli shift from negative (poaching) to neutral (inside the Reserve, without poaching).

Our results show, similarly to other studies, that ungulates can reduce predation risk by forming groups [45], we found larger groups in the UA than in DCR. Probably, as proposed by Taraborelli and collaborators [17], the benefit from living in groups for the guanaco in our study area is due to cooperative vigilance. Moreover, unlike Stephens and Peterson [19], who found that in moose the groups with offspring are more vulnerable than groups without them [19], we did not find a differential effect of poaching on each group category. We found a similar proportion for each group category in both areas.

Environmental factors also have a significant influence on ungulate behavior [10, 13]. In relation to habitat structure, similarly to Taraborelli and collaborators [13], we found that neither FID nor group size were affected by slope, probably due to the great ability of guanacos to climb. On the other hand, vegetation cover was not important for group size but it was an important factor in modulating the risk assumed by guanacos, since our results show a low FID (high risk) in areas with high vegetation cover. For the same population, it was found that guanacos use vegas (sites with high vegetation quality) even if they entail high predation risk [15]. Probably, due to the energy benefits gained in areas with high vegetation cover, the guanaco could stay in areas with greater food availability despite a high predation risk. Different aspects of foraging behavior (i.e. food items selected, selection of sites with high vegetation quality) deserve further research in order to assess this hypothesis.

According to our results, the guanaco population in this cold desert responds to poaching pressure as do other species of ungulates. Most of the groups occurred in the protected area and showed higher FID and larger group size compared to the unprotected area. For this population, FID can be a useful tool for evaluating the effect of poaching on guanacos, and how they respond to this human disturbance. In addition, managers must take habitat structure into account, since the guanaco appears to assume a higher risk (lower FID) in areas with high vegetation cover.

## Supporting information

S1 Table (XLSX)
